# Complex abdominal wall reconstruction after massive resection due to neoplastic invasion: a case report

**DOI:** 10.1093/jscr/rjab342

**Published:** 2021-08-16

**Authors:** Rodrigo Piltcher-da-Silva, Débora Oliveira Hütten, Lucas Dorídio Locks-Coelho, Mariana Piltcher-Recuero, Bernardo Silveira Volkweis, Cláudio Tarta, Márcia Luiza Appel, Leandro Totti Cavazzola

**Affiliations:** General Surgery Service, Hospital de Clínicas de Porto Alegre, Universidade Federal do Rio Grande do Sul (UFRGS), Porto Alegre, Brazil; General Surgery Service, Hospital de Clínicas de Porto Alegre, Universidade Federal do Rio Grande do Sul (UFRGS), Porto Alegre, Brazil; Obstetrics and Gynecology Service, Hospital de Clínicas de Porto Alegre, Universidade Federal do Rio Grande do Sul (UFRGS), Porto Alegre, Brazil; General Surgery Service, Hospital de Clínicas de Porto Alegre, Universidade Federal do Rio Grande do Sul (UFRGS), Porto Alegre, Brazil; General Surgery Service, Hospital de Clínicas de Porto Alegre, Universidade Federal do Rio Grande do Sul (UFRGS), Porto Alegre, Brazil; Coloproctology Service, Hospital de Clínicas de Porto Alegre, Universidade Federal do Rio Grande do Sul (UFRGS), Porto Alegre, Brazil; Obstetrics and Gynecology Service, Hospital de Clínicas de Porto Alegre, Universidade Federal do Rio Grande do Sul (UFRGS), Porto Alegre, Brazil; General Surgery Service, Hospital de Clínicas de Porto Alegre, Universidade Federal do Rio Grande do Sul (UFRGS), Porto Alegre, Brazil

## Abstract

Complex reconstructions of the abdominal wall, necessary after resection of neoplasms, infection or trauma, are a challenge for the surgical team. Although ovarian carcinoma is commonly presented with peritoneal carcinomatosis and invasion of adjacent organs, it rarely can invade the abdominal wall. Invasion of the abdominal wall was documented on ultrasound and abdominal computed tomography. Surgery was discussed and performed in a multidisciplinary team and consisted of wide *en bloc* excision and reconstruction with open intraperitoneal onlay mesh with inorganic polypropylene-coated mesh (Bard/BD Sepramesh), a midweight macroporous mesh and abdominoplasty. Postoperative course was uneventful and the patient showed good evolution 1 year after the procedure. Our report highlights the main objectives in complex reconstructions, the importance of a multidisciplinary team and discusses the characteristics that the mesh must have in order to achieve the desired goal.

## INTRODUCTION

Complex abdominal wall (AW) reconstructions must aim tissue coverage of the defect and the least possible loss of musculoaponeurotic functionality, and represent a challenge for the surgical team [1, 2]. The causes of these major defects are tumors, infections and trauma.

For adequate coverage of the defect and to add strength to AW, many reconstruction techniques are available and vary between flaps, mesh and larger surgeries, such as component separation [2, 3]. The use of mesh gained space due to the need for greater mechanical and dynamic strength in the reconstruction of the AW [1]. However, it is still under discussion which is the most appropriate one.

A case of a patient with high-grade serous carcinoma (HGSC) with invasion of the AW up to 4 mm from the skin and with indication for wide excision and reconstruction will be discussed here. Among the cases of AW reconstruction found in the literature, few clearly report the type of mesh used, which is a determining factor for success.

## PRESENTATION OF CASE

**Figure 1:**
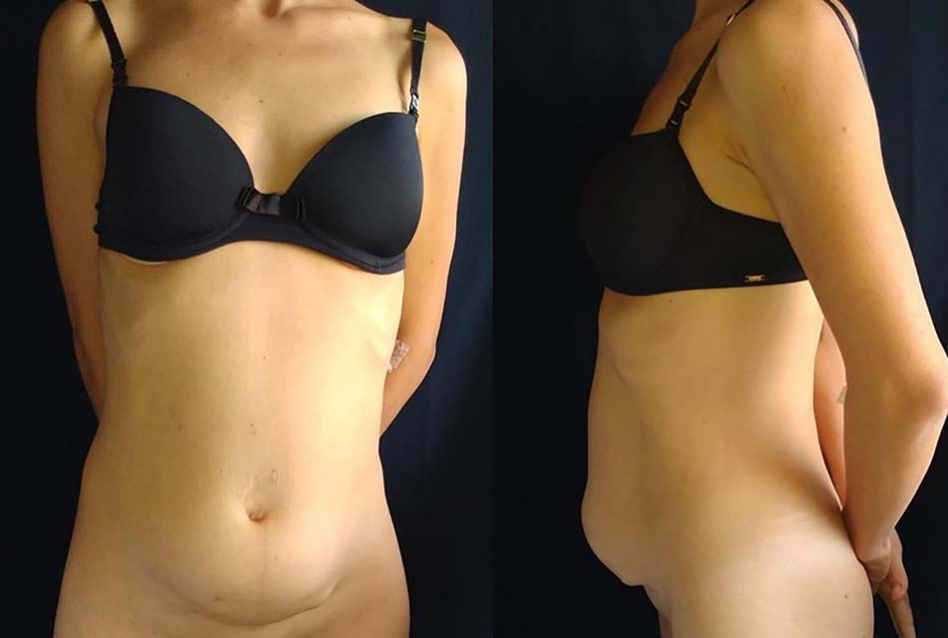
Abdominal wall with tumor invasion and bulging.

Female patient, age 35, with a previous diagnosis of nephrotic syndrome. In March 2019, 8 months after a cesarean delivery, the patient complained of painful subcutaneous nodules in the abdomen, amenorrhea and feeling abdominal bloating. Upon examination, nodules fixed to the deep fascia were palpable in the hypogastrium with local burning pain. The patient arrived at the referral hospital in November 2019, when nodule growth was identified (Fig. 1).

Examinations showed an increase in cancer antigen 125 (CA-125) from 73 U/ml in May to 929.7 U/ml in December 2019. Ultrasound showed coalescent nodules affecting the rectus abdominis muscle bilaterally in the infraumbilical region, extending to the subcutaneous with a superficial limit of 0.4 cm from the skin. Transvaginal ultrasound showed 2.1 × 1.1 cm ovarian parenchyma on the right and, in contiguity, an expansive lesion of heterogeneous content, irregular walls and anechoic cystic area. At Doppler, low impedance rates (0.3) were found.

**Figure 2:**
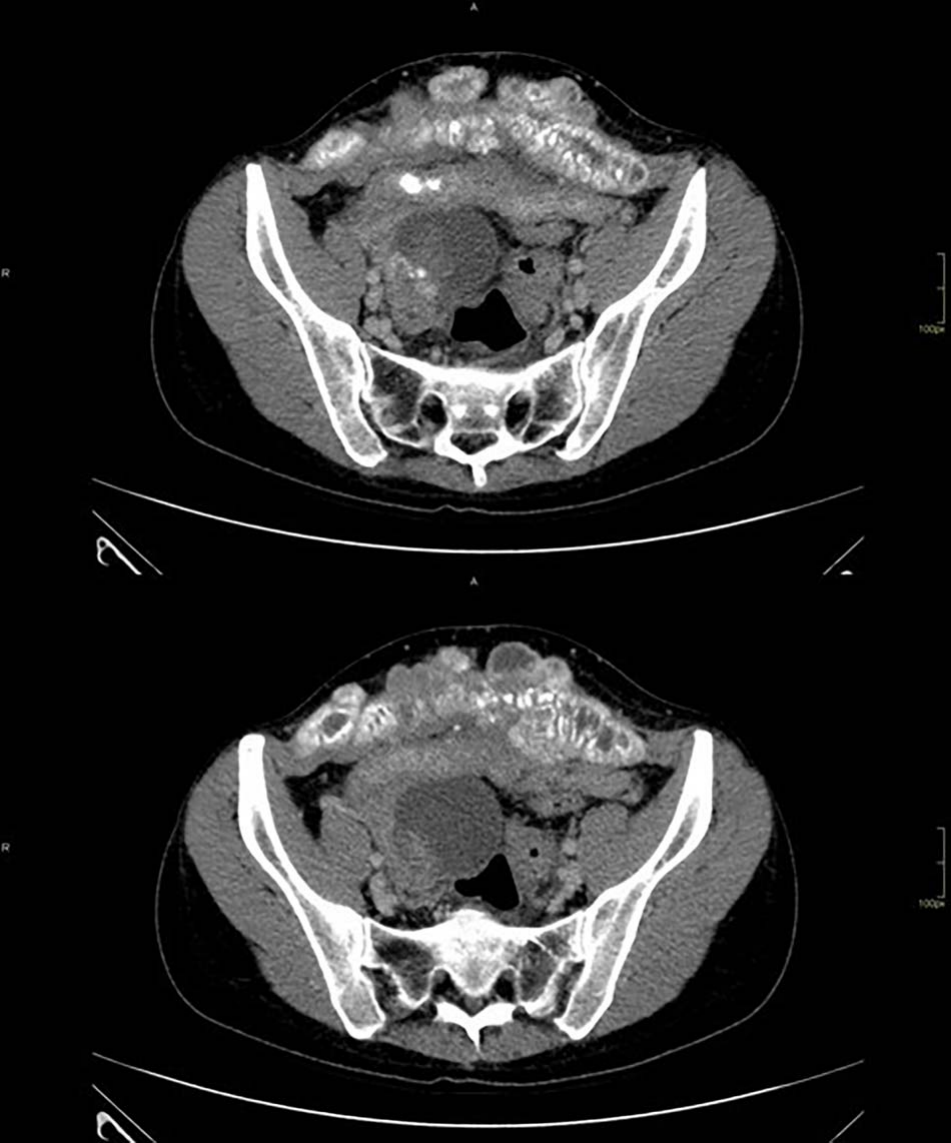
CT with heterogeneous solid-cystic lesions with contrast enhancement, septations and gross calcifications, in the topography of the uterine attachments, measuring 5.8 × 4.9 × 5.0 cm on the right (74 cm^3^ volume), 7.8 × 8.7 × 7.9 cm (278 cm^3^ volume) in the region of the rectouterine pouch and 2.9 × 2.4 × 2.3 cm (8.3 cm^3^ volume) in the left adnexal region.

**Figure 3:**
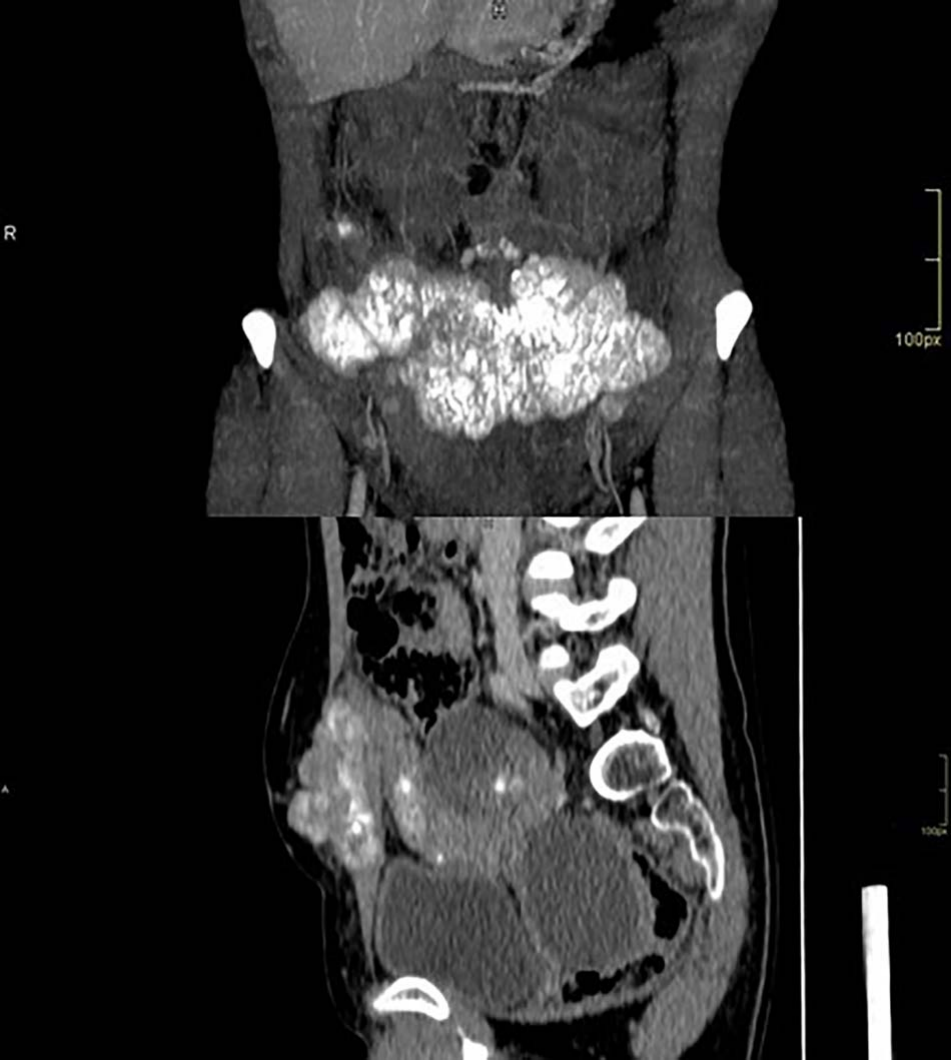
Abdominal wall CT with expansive lesion with soft tissue density, heterogeneous, with the appearance of a cluster of nodules, measuring 16.4 × 5.1 × 7.7 cm (334 cm^3^ volume) in the major latero-lateral, antero-posterior axes and longitudinal, respectively.

Computed tomography of the abdomen showed solid-cystic lesions in the pelvis (Fig. 2) and a massive expansive lesion in the AW, with soft tissue density and a volume of 334 cm^3^ (Fig. 3). Between the AW and the pelvic lesions, formation with soft tissue density, heterogeneous enhancement and calcifications are identified, measuring 12.5 × 2.5 cm in the major latero-lateral and anteroposterior axes, compatible with ‘omental cake’.

Ultrasound-guided biopsy was performed, with anatomopathological and immunohistochemistry of low-grade serous carcinoma of genital tract; referred to oncology for neoadjuvant chemotherapy (carboplatin plus paclitaxel). There was a severe reaction to paclitaxel and a surgical approach was chosen. The gynecological–oncology, general and plastic surgery teams opted for *en bloc* resection.

**Figure 4:**
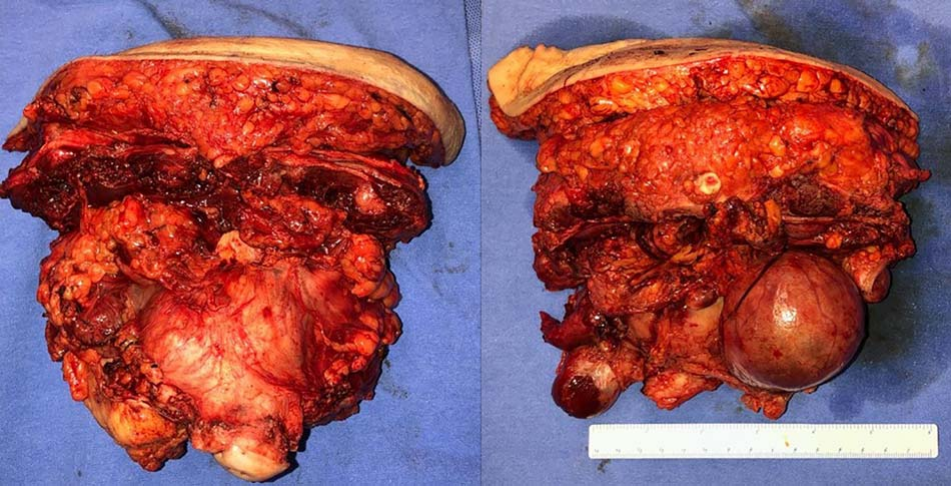
Surgical specimen of pan-hysterectomy with excision of the abdominal wall and cecectomy.

**Figure 5:**
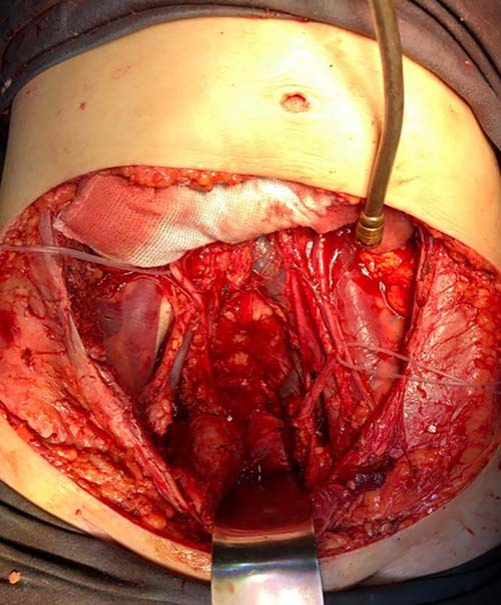
Pelvis after tumor excision, peritonectomy and lymphadenectomy.

**Figure 6:**
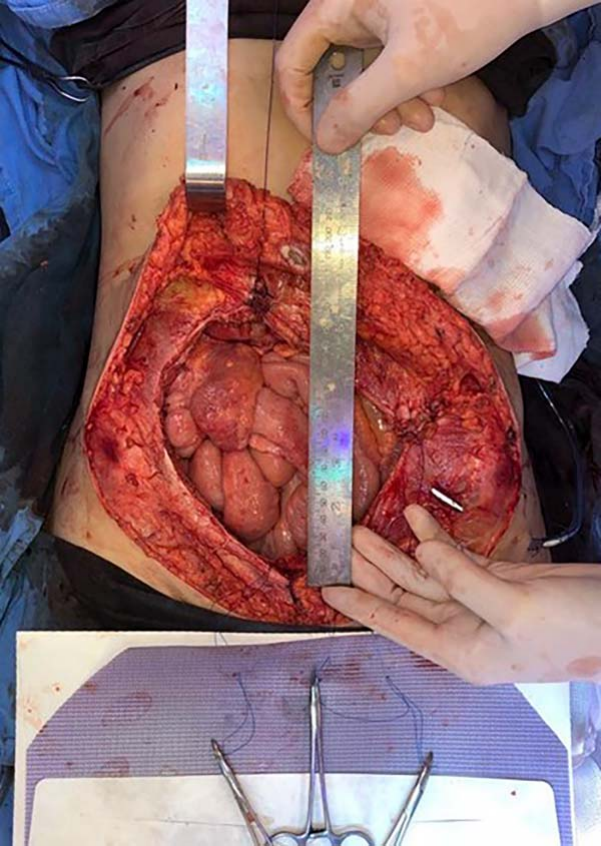
Defect of the abdominal wall of 7 × 7 cm in the major axes.

**Figure 7:**
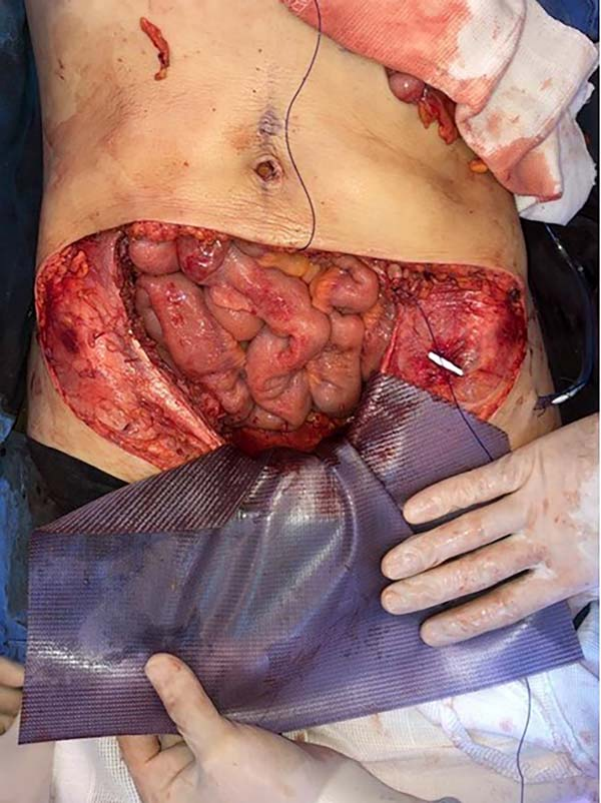
Mesh initially fixed on the pubic tubercle and Cooper’s ligament bilaterally.

**Figure 8:**
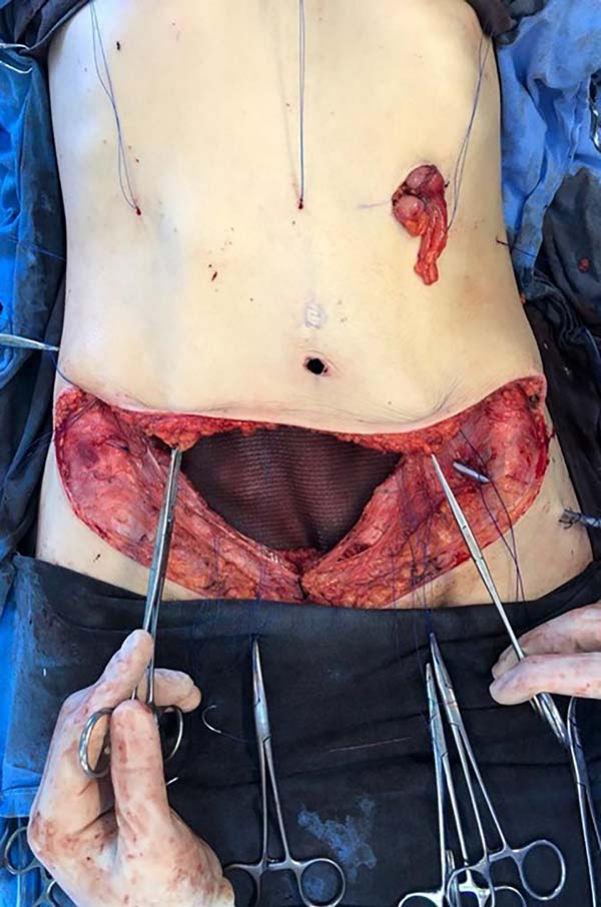
Mesh covering the abdominal defect with presentation of transfascial fixation points, with an overlap of 8 cm.

**Figure 9:**
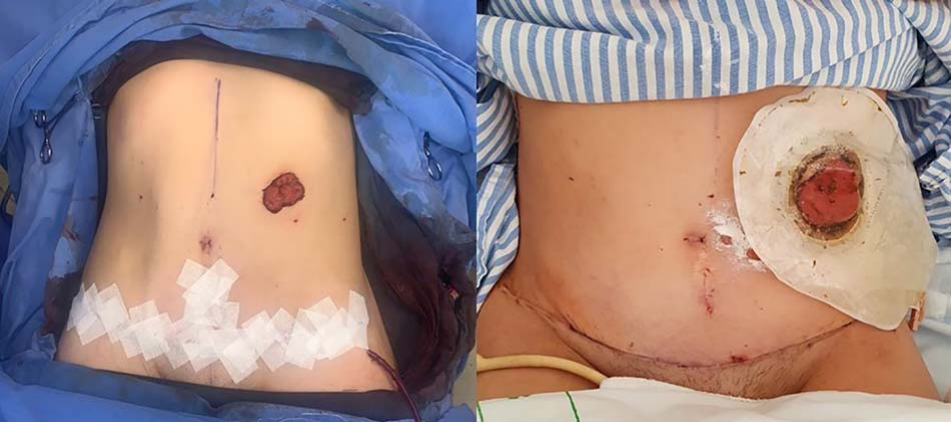
Final image of the surgery.

**Figure 10:**
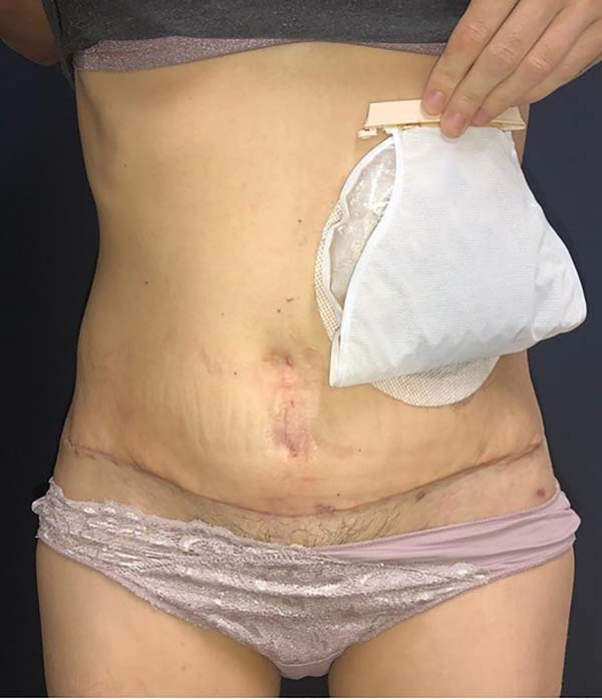
Appearance after 1 year of surgery.

In April 2020, the surgery was performed, with pan-hysterectomy, excision of the AW and skin, pelvic peritonectomy, cecectomy (due to local involvement), partial cystectomy, Hartmann rectosigmoidectomy (Figs 4 and 5), followed by AW partial closure and an intraperitoneal onlay mesh (Open IPOM) in a bridged position with Bard Mesh/BD Sepramesh. Abdominoplasty was performed to allow skin coverage (Figs 6–10). The patient stayed in hospital for 18 days and presented urinary retention. Definitive anatomopathological was HGSC, infiltrating uterus, AW, cecum, rectosigmoid and obturator lymph node metastasis. After cytoreduction, she underwent chemotherapy (carboplatin plus docetaxel).

In 1 year of follow-up, the patient presented good healing and adequate adaptation to surgery (Fig. 10), complicated only by the hypoactivity of the urinary bladder—requiring intermittent catheterization and prophylactic nitrofurantoin. However, the disease progressed in February 2021. Due to the low volume of metastatic disease, absence of symptoms and low tolerance to chemotherapy, it was decided to start treatment only if clinical progression occurs.

## DISCUSSION

AW reconstruction after massive resections can be achieved with several strategies.

Latissimus dorsi and anterolateral pedicle flap are used with acceptable local morbidity, but a 40% risk of hernia occurrence [3, 4]. The high rate of hernia is due to the low resistance of the tissue, a complication that must be avoided due to the impact on quality of life [1].

To add strength and resistance to reconstruction, a polypropylene synthetic, non-absorbable and resistant mesh, seems to be the standard [1, 2, 4]. There are concerns about complications such as infection, chronic pain, intestinal adhesion, fistula and obstruction, and to reduce the risk, the biological and biosynthetic mesh were developed [5, 6]. However, they were not superior to the non-absorbable synthetic in the presence of contamination, and when used as a bridge, high levels of postoperative hernia (56–80%) were found [2, 7, 8].

We used Bard/BD Sepramesh surgical mesh (101 g/m^2^ polypropylene, 234 g/m^2^ total, pore: 0.35 mm^2^) because it is macroporous midweight and with non-stick coating for contact with the intestine, ensuring strength, good integration and protection for contact with the intestinal loops. The coating with bioresorbable hydrogel provides security for contact with the loops, reducing the risk of adhesions and fistulas, inherent to polypropylene.

Macroporous midweight mesh appears to have better results for strength and tissue integration, whereas lightweight mesh has a higher risk of hernia and central failure. Microporous heavyweight has less resistance to infection, without adding significant strength [1, 9]. For IPOM use, common in complex reconstructions, midweight mesh is considered the best choice.

To seek better AW functionality, fixation is crucial when the mesh is positioned like in this case, performed at symmetrical points in the remaining abdominal rectus aponeurosis, on the lateral AW, pubis and bilateral cooper ligament, in addition to a minimum 5 cm overlap to reduce the risk of hernia [10]; thus, properly distributing the tension force on the AW.

Several surgical techniques are used in AW reconstructions and the mesh seems to be indispensable if the objective is to improve functionality and mechanical strength. Biological, biosynthetic and absorbable fabrics failed to overcome polypropylene, as expected. In summary, our report highlights a successful complex AW reconstruction, and we believe that larger studies comparing reconstruction techniques and the use of mesh should be conducted to better understand this complex surgery.
